# Protocol for double-crosslinking ChIP-seq to improve data quality and enhance detection of challenging chromatin targets

**DOI:** 10.1016/j.xpro.2025.104171

**Published:** 2025-10-29

**Authors:** Yu Bao, Roberta Cacioppo, Alastair Crisp, Ivan Shlamovitz, Julian E. Sale, Ana Tufegdzic Vidakovic, Stephin J. Vervoort, Andrew Zeller

**Affiliations:** 1 MRC Laboratory of Molecular Biology, Cambridge CB2 0QH, UK; 2 The Walter and Eliza Hall Institute of Medical Research, Parkville, VIC 3052, Australia

**Keywords:** Genomics, Molecular Biology, Chromatin immunoprecipitation, ChIP

## Abstract

Chromatin factors drive genome function, yet many lack direct DNA-binding activity and cannot be conventionally profiled. Here, we present dxChIP-seq, a double-crosslinking chromatin immunoprecipitation sequencing (ChIP-seq) protocol that improves mapping of chromatin factors, including those that do not bind DNA directly, while enhancing signal-to-noise ratio. We describe steps for double-crosslinking, focused ultrasonication, immunoprecipitation, DNA purification, and library preparation. We then detail procedures for sequencing and analysis. This protocol is compatible with adherent cells and complex multicellular structures.

For complete details on the use and execution of this protocol, please refer to Cacioppo et al.^1^

## Highlights

• Steps for dual-crosslinking capture proteins directly and indirectly bound to DNA
• Instructions for chromatin extraction and focused ultrasonication
• Procedures for immunoprecipitation and DNA purification
• Guidance on sequencing, data processing, and normalization strategies

## Abstract (editor highlights)

Publisher’s note: Undertaking any experimental protocol requires adherence to local institutional guidelines for laboratory safety and ethics.

## Graphical abstract

**Figure:**
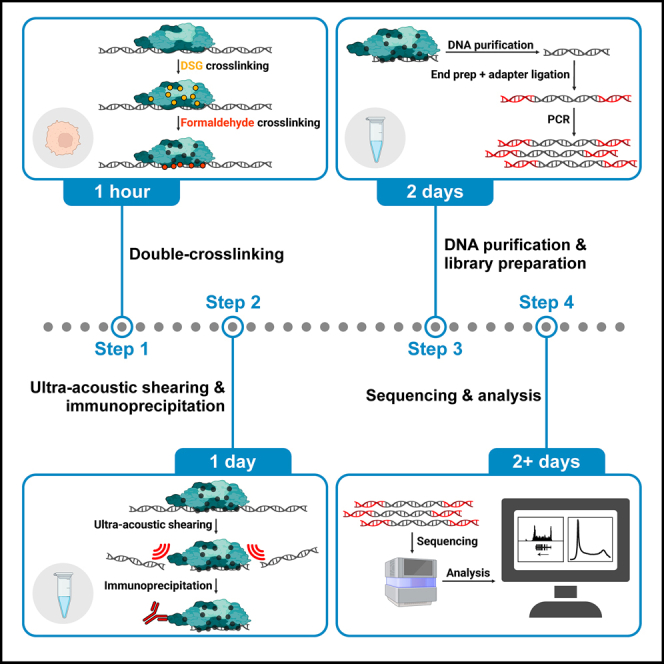

## Before you begin

Chromatin factors are pivotal regulators of genome function. These proteins include general transcription machinery, chromatin remodelers, histone modifiers, and replication and repair complexes. Together, they coordinate transcriptional programs and maintain genome integrity through dynamic interactions with chromatin. Misregulation is associated with developmental disorders, cancer, and other human diseases. Mapping the genome-wide occupancy of chromatin factors is therefore essential for understanding gene regulation and genome maintenance.

**Figure 1:**
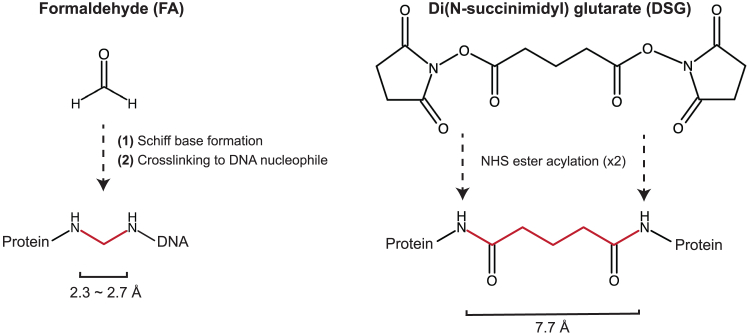
Comparison of FA and DSG crosslinking chemistries Formaldehyde typically crosslinks proteins to DNA through a two-stage process: it first reacts with a protein nucleophile to form a reversible intermediate, then reacts with a DNA nucleophile to yield a short methylene bridge (∼2.3–2.7 Å). In contrast, disuccinimidyl glutarate (DSG) forms protein–protein crosslinks through two independent ester acylation reactions that do not generate DNA-reactive intermediates, resulting in stable amide bonds across a longer spacer (∼7.7 Å).

The most widely used approach, ChIP-seq, relies on the crosslinking chemistry of formaldehyde (FA). FA is a small electrophilic aldehyde that reacts primarily with nucleophilic sites in proteins – most often the ε-amino group of lysine side chains, but also other residues such as arginine, histidine, and cysteine.^2^ At physiological pH, lysine residues are mostly protonated and positively charged, naturally positioning them near the negatively charged DNA backbone in DNA-binding proteins. A small unprotonated fraction remains nucleophilic and able to react with FA. Crosslinking proceeds in two steps. First, FA reacts with a nucleophile to form a reactive intermediate, such as a Schiff base or hydroxymethyl adduct; this can then couple to a second nucleophile, including the exocyclic amino groups of DNA bases, to form a very short (∼2 Å) methylene bridge^3^ (Figure 1). Because lysine residues are often positioned close to DNA, this sequential, zero-length chemistry strongly favors protein-DNA crosslink formation.

The same chemistry makes FA less effective at capturing protein–protein associations. To link two proteins, FA must first react with a nucleophilic site on one residue, then couple to a second nucleophile within ∼2 Å – a spacing less reliably achieved at the looser interfaces typical of protein-protein contacts. Because ChIP-seq requires crosslinks to be reversible for DNA recovery, the efficiency of protein-protein capture cannot simply be increased by raising FA concentration or exposure time. Instead, protocols use mild and reversible conditions – typically 1% FA for ∼10 min at room temperature – which generate crosslinks that can be cleaved by prolonged heating (∼65°C for several hours). These constraints limit protein-protein crosslinking and stabilization, leading to underrepresentation of indirectly bound factors and multi-protein complexes. Since many chromatin regulators act through such assemblies, standard ChIP-seq fails to capture a large subset of physiologically relevant proteins.

To address this limitation, we developed a double-crosslinking ChIP-seq protocol (dxChIP-seq; introduced by Cacioppo et al.,^1^ building on Tufegdzic Vidakovic et al.^4^) that incorporates disuccinimidyl glutarate (DSG) in the first step to stabilize protein complexes and indirectly bound targets. DSG is a homobifunctional NHS-ester crosslinker, with two reactive esters joined by a five-atom glutarate spacer^5^ (∼7.7 Å). Unlike the zero-length chemistry of FA, this spacer matches distances typical of protein–protein interfaces. Each NHS ester independently acylates a primary amine, generally at lysine residues, forming stable amide bonds at both ends without generating DNA-reactive intermediates (Figure 1). Thus, its defined spacer and non-sequential chemistry efficiently stabilize protein assemblies while contributing little to protein–DNA crosslinking. Sequential use of DSG and FA is therefore complementary: DSG first ‘locks’ protein–protein contacts, and FA then secures protein–DNA interactions, together providing a more complete capture of protein complexes on DNA.

### Innovation

**Figure 2:**
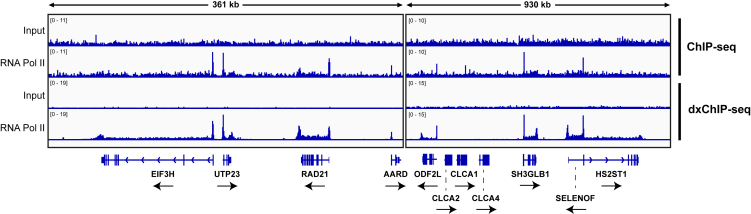
Comparison of ChIP-seq and dxChIP-seq performance at low-occupancy genes RPKM-normalized IGV tracks from ChIP-seq and dxChIP-seq experiments targeting RNA Pol II (D8L4Y antibody) in wild-type HEK-293 cells. dxChIP-seq was performed using the protocol described in this paper, while classical ChIP-seq was performed under standard conditions.^8^

Our key innovation lies in re-optimizing the ChIP-seq protocol to exploit the complementary chemistries of DSG and FA crosslinking. We systematically refined crosslinking, lysis, and shearing conditions – identifying effective parameters for DSG and FA crosslinking, buffer composition, chromatin concentration during shearing, and ultrasonication settings (see details in the main text). In contrast to earlier studies,^5^^,^^6^^,^^7^ we found that relatively short crosslinking times (1.66 mM DSG for 18 min, followed by 1% FA for 8 min at room temperature) strike the best balance between preserving chromatin architecture and avoiding over-fixation. Optimized ultrasonication achieves efficient fragmentation without compromising the integrity of crosslinked protein–DNA complexes. Together, these refinements enhance detection of chromatin factors, particularly at low-occupancy regions that are difficult to capture with standard protocols (Figure 2), and broaden the range of proteins amenable to ChIP-seq. In our experience, dxChIP-seq has proven effective for probing RNA Pol II, the Mediator complex, the PAF complex, and histone modifications (unpublished data).

## Key resources table

**Table:** 

REAGENT or RESOURCE	SOURCE	IDENTIFIER
**Antibodies**		

Rabbit monoclonal RPB1 (total, N-terminal) (8 μg per IP)	Cell Signaling Technology	D8L4Y; RRID:AB_2687876
Spike-in antibody (1–2 μg per IP)	Active Motif	61686
Rabbit anti-rat IgG H&L (40 μg per IP)	Abcam	ab6703

**Biological samples**		

Spike-in chromatin	Active Motif	53083

**Chemicals, peptides, and recombinant proteins**		

16% formaldehyde (w/v), methanol-free	Thermo Scientific	28908
DSG (disuccinimidyl glutarate)	Thermo Scientific	20593
PBS	N/A	N/A
DMSO	Sigma-Aldrich	D8418
HEPES-KOH pH 7.5	N/A	N/A
Tris-HCl pH 7.5	N/A	N/A
Tris-HCl pH 8	N/A	N/A
NaCl	N/A	N/A
Glycerol	N/A	N/A
EDTA, pH 8	N/A	N/A
LiCl	VWR	0416
EGTA, pH 8	Millipore	324626
Sodium dodecyl sulfate (SDS)	Sigma-Aldrich	436143
Sodium deoxycholate	Sigma-Aldrich	SRE0046
N-lauroylsarcosine sodium salt	Sigma-Aldrich	L5125
Triton X-100	Sigma-Aldrich	93443
NP-40 alternative	Millipore	492016
Proteinase K	Invitrogen	AM2546
Bovine serum albumin (BSA)	Merck	A9418
cOmplete protease inhibitor cocktail	Roche	11697498001
PhosSTOP phosphatase inhibitor cocktail	Sigma-Aldrich	04906837001
N-ethylmaleimide (NEM)	Sigma-Aldrich	04260-5G-F
Nuclease-free water	Invitrogen	AM9937
PBS, pH 7.4	Gibco	10010
Glycine	Millipore	104201
RNase A	Invitrogen	12091021

**Critical commercial assays**		

Qubit dsDNA high sensitivity assay kit	Invitrogen	Q33230
Protein G Dynabeads	Fisher Scientific	10004D
ChIP DNA Clean & Concentrator	Zymo Research International	D5205
NEBNext Ultra II DNA library prep kit	NEB	E7645L
NextSeq 2000 P3 XLEAP-SBS reagent kit (100 cycles)	Illumina	20100990
Agilent Bioanalyzer high sensitivity DNA kit	Agilent	5067-4626
Agilent D1000/D5000 ScreenTape	Agilent	5067-5582/5067-5588

**Deposited data**		

dxChIP-seq data	Cacioppo et al.^1^	GEO: GSE276375

**Experimental models: Cell lines**		

Flp-In T-Rex HEK-293 cells	Thermo Fisher Scientific	R78007

**Oligonucleotides**		

NEBNext multiplex oligos for Illumina (96 unique dual index primer pairs set 3)	NEB	E6444S

**Software and algorithms**		

Trim Galore v.0.6.7	Babraham Bioinformatics	https://github.com/FelixKrueger/TrimGalore
Bowtie2 v.2.5.1	Langmead et al.^9^; Langmead and Salzberg^10^	https://github.com/BenLangmead/bowtie2
SAMtools v.1.9	Li et al.^11^	https://www.htslib.org/
Picard v.2.27.5	Broad Institute	http://broadinstitute.github.io/picard
DeepTools v.3.5.1	Ramírez et al.^12^	https://github.com/deeptools/deepTools
BEDtools v.2.30.0	Quinlan and Hall^13^	https://bedtools.readthedocs.io/en/latest/
dplyr	Wickham et al.^14^	https://cran.r-project.org/web/packages/dplyr/index.html
rtracklayer	Lawrence et al.^15^	https://bioconductor.org/packages/release/bioc/html/rtracklayer.html
ggplot2	Wickham^16^	https://cran.r-project.org/web/packages/ggplot2/index.html

**Other**		

Tissue culture dishes (15 cm)	N/A	N/A
50 mL Falcon tubes	N/A	N/A
15 mL Falcon tubes	N/A	N/A
Cell scrapers	N/A	N/A
Centrifuge suited for 15 mL conical tubes, with refrigeration	N/A	N/A
Liquid nitrogen	N/A	N/A
Laminar flow hood bench	N/A	N/A
Chemical hood	N/A	N/A
Covaris E220 focused ultrasonicator	Covaris	E220
Covaris milliTUBE 1 mL AFA fiber	Covaris	520135
Protein LoBind tubes 2 mL	Eppendorf	Z666513
Magnetic rack to hold 2 mL tubes	N/A	N/A
Bioanalyzer for DNA analysis	N/A	N/A
NextSeq 2000 sequencing system	Illumina	N/A
Agilent 2100 Bioanalyzer system	Agilent	N/A
Agilent 4200 TapeStation system	Agilent	N/A

## Materials and equipment

### Antibodies

Antibody selection is critical for dxChIP-seq success, as the antibody must recognize its target selectively and remain effective despite fixation-induced modifications. Antibodies previously used in ChIP-seq are ideal, though those effective in techniques using other forms of fixation (IHC-P, immunocytochemistry, flow cytometry, etc.) can also be considered. A quick way to screen potential antibodies is to check their specificity on formaldehyde-fixed samples by immunoprecipitation (IP) and western blot. Samples treated with DSG are not useful for this purpose, as protein-protein crosslinks are irreversible and alter the electrophoretic mobility of the protein. In contrast, formaldehyde-fixed samples provide a good indication of antibody performance in dxChIP-seq.

### Next-generation sequencing

The protocol described here produces libraries compatible with Illumina sequencing platforms, though it can be adapted to generate libraries for MGI sequencing if required. We typically use the NextSeq2000 System with XLEAP-SBS reagents, sequencing in paired end mode with a minimum of 100 cycles. The desired number of reads depends on the target protein and number of samples (see step 58). We do not recommend the use of longer-read sequencing due to the nature of the samples generated.

### Chromatin shearing

Many chromatin shearing devices have been used for ChIP-seq, including small-volume probe sonicators, Diagenode Bioruptors, Active Motif PIXUL, and Covaris focused-ultrasonicators. In our hands, the Covaris system yields the most reproducible results, minimizing protein degradation and denaturation while fragmenting DNA to an ideal size. The fragmentation procedure described herein uses a Covaris E220, with parameters optimized for HEK-293 cells. If using an alternative cell line, protein or fragmentation device, these parameters may require adjustment. See troubleshooting – problem 1 for guidance on optimizing fragmentation; if using a different sonication device, use the manufacturer’s recommended settings as a starting point.

### Glycine solution (1.25 M)

Prepare 20 mL 1.25 M glycine solution by dissolving 1.88 g glycine in 15 mL PBS, making sure the powder is fully dissolved. Adjust the final volume to 20 mL with PBS, then sterile-filter the solution using a syringe-driven 0.22 μm filter unit. Store at room temperature and use on the same day.

### NEM (200 mM)

Prepare 1 mL of 200 mM NEM solution by weighing 25 mg of NEM powder in a 2 mL tube. Add 1 mL of ethanol and vortex until the NEM is dissolved. Store on ice and use on the same day.

### Sodium deoxycholate (10%)

Prepare 40 mL 10% of sodium deoxycholate solution by dissolving 4 g of sodium deoxycholate powder in 30 mL of Milli-Q water in a 50 mL Falcon tube. Gently rock the tube in the dark at 4°C until the powder is fully dissolved. Adjust the final volume to 40 mL with Milli-Q water, then sterile-filter the solution using a syringe-driven 0.22 μm filter unit. The solution can be stored in the dark at 4°C for up to 1 year.

### FA dilution buffer

Store at room temperature for up to 6 months.

**Table:** 

Reagents	Final concentration	Amount
1 M HEPES-KOH, pH 7.5	50 mM	25 mL
5 M NaCl	100 mM	10 mL
0.5 M EDTA, pH 8	1 mM	1 mL
0.5 M EGTA, pH 8	0.5 mM	0.5 mL
Milli-Q water	N/A	463.5 mL
**Total**	–	**500 mL**

### LB1 buffer

Store at room temperature for up to 6 months. Add protease inhibitor cocktail (1 tablet per 50 mL), phosphatase inhibitor (1 tablet per 10 mL) and NEM (final concentration 2 mM) on the day of use.

**Table:** 

Reagents	Final concentration	Amount
1 M HEPES-KOH, pH 7.5	50 mM	25 mL
5 M NaCl	140 mM	14 mL
0.5 M EDTA, pH 8	1 mM	1 mL
Glycerol	10% (v/v)	50 mL
NP-40 alternative	0.5% (v/v)	2.5 mL
Triton X-100	0.25% (v/v)	1.25 mL
Milli-Q water	N/A	∼406.25 mL
**Total**	–	**500 mL**

### LB2 buffer

Store at room temperature for up to 6 months. Add protease inhibitor cocktail (1 tablet per 50 mL), phosphatase inhibitor (1 tablet per 10 mL) and NEM (final concentration 2 mM) on the day of use.

**Table:** 

Reagents	Final concentration	Amount
1 M Tris-HCl, pH 8	10 mM	5 mL
5 M NaCl	200 mM	20 mL
0.5 M EDTA, pH 8	1 mM	1 mL
0.5 M EGTA, pH 8	0.5 mM	0.5 mL
Milli-Q water	N/A	∼473.5 mL
**Total**	–	**500 mL**

### LB3 buffer

Store at room temperature for up to 6 months. Add protease inhibitor cocktail (1 tablet per 50 mL), phosphatase inhibitor (1 tablet per 10 mL) and NEM (final concentration 2 mM) on the day of use. Add sodium deoxycholate (final concentration 0.1%) directly before use.

**Table:** 

Reagents	Final concentration	Amount
1 M Tris-HCl, pH 8	10 mM	0.5 mL
5 M NaCl	100 mM	1 mL
0.5 M EDTA, pH 8	1 mM	0.1 mL
0.5 M EGTA, pH 8	0.5 mM	0.05 mL
N-Lauroylsarcosine	0.5% (w/v)	0.25 g
Milli-Q water	N/A	∼48.35 mL
**Total**	–	**50 mL**

### BSA-PBS solution

Prepare BSA-PBS solution freshly before using.

**Table:** 

Reagents	Final concentration	Amount
PBS, pH 7.4	1×	100 mL
Bovine serum albumin	0.1% (w/v)	0.1 g
**Total**	–	**100 mL**

### 2× decrosslinking buffer

Store at room temperature for up to 6 months.

**Table:** 

Reagents	Final concentration	Amount
1 M Tris-HCl, pH 8	10 mM	0.5 mL
5 M NaCl	200 mM	2 mL
0.5 M EDTA, pH 8	10 mM	1 mL
10% (v/v) SDS	0.5% (v/v)	2.5 mL
Milli-Q water	N/A	∼44 mL
**Total**	–	**50 mL**

### RIPA buffer

Prepare RIPA freshly before using.

**Table:** 

Reagents	Final concentration	Amount
1 M HEPES-KOH, pH 7.5	50 mM	25 mL
5 M LiCl	500 mM	50 mL
0.5 M EDTA, pH 8	1 mM	1 mL
NP-40 alternative	1% (v/v)	5 mL
Sodium deoxycholate (10%)	0.7% (w/v)	35 mL
Protease inhibitor cocktail	1×	1 tablet per 50 mL
Milli-Q water	N/A	∼384 mL
**Total**	–	**500 mL**

### ChIP elution buffer

Prepare ChIP elution buffer freshly before using.

**Table:** 

Reagents	Final concentration	Amount
1 M Tris-HCl, pH 7.5	25 mM	1.25 mL
0.5 M EDTA, pH 8	5 mM	0.5 mL
10% (v/v) SDS	0.5% (v/v)	2.5 mL
Milli-Q water	N/A	∼45.75 mL
**Total**	–	**50 mL**

## Step-by-step method details

### Prepare cells for crosslinking

**Timing: 1 day**

This step describes the recommended conditions for seeding cells.

1. Seed cells so that they reach ∼80% confluency at the time of crosslinking.

***Note:*** Aim for ∼40 million cells per condition, per antibody, per biological replicate. For HEK-293 cells, this typically involves seeding 2 × 15 cm dishes per IP, each with 8–10 million cells, ensuring even cell distribution across the dish surface for optimal crosslinking efficiency. After overnight growth, this yields ∼20 million cells per dish. Depending on the number of protein targets, we generally recommend seeding cells for each condition and replicate in batches of 6 × 15 cm dishes – enough for parallel IPs using three different antibodies.

**CRITICAL:** We recommend generating three biological replicates per condition. Seed each replicate on different days, or from different splits of the same culture, but ensure that all samples are otherwise treated identically.

***Note:*** If material is limited, substantially lower inputs can be used. When using high quality antibodies, we have successfully performed dxChIP-seq with as few as 8 million cells per IP. If working with low cell numbers, fewer than three protein targets, different cell lines, organoids or tissues, ensure that the amount of lysis and shearing buffers are scaled proportionally to cell number in later steps.

### Perform double-crosslinking *in vivo*

**Timing: 1–3 h. Duration is dependent on the number of experimental conditions**

This section describes the double crosslinking procedure for HEK-293 cells seeded on 15 cm tissue culture dishes. If rapid processing is required (e.g. due to precise timing of cell treatments), consult your institutional health and safety department to verify if crosslinking steps can be carried out in a laminar flow hood, following the steps below to minimize evaporation of formaldehyde. Alternatively, use a fume hood.

2. Prepare FA dilution buffer and 1.25 M glycine solution; keep at room temperature until use.
3. Pre-chill PBS on ice and set the centrifuge temperature to 4°C.
4. Set up the working station under a laminar flow hood / fume hood by gathering:
  a. Freshly prepared FA dilution buffer, glycine solution and 1.66 mM DSG aliquots.
  b. 16% or 37% methanol-free formaldehyde solution.
  c. Room temperature PBS.
  d. A bucket of ice with pre-chilled PBS next to the workstation.
  e. 15 mL Falcon tubes for collecting cells after crosslinking, labeled in advance.
  f. A 1 L beaker for general liquid waste.
  g. A separate container with a lid for formaldehyde-contaminated liquid waste.
5. Freshly prepare 0.5 M DSG in anhydrous DMSO, then dilute to 1.66 mM in PBS.

**CRITICAL:** Allow frozen DSG powder to equilibrate to room temperature before opening to prevent moisture absorption from the air. Both 0.5 M and 1.66 mM DSG solutions should be prepared immediately before use.

***Note:*** Prepare 12 mL of 1.66 mM DSG solution per 15 cm tissue culture dish. To streamline handling, aliquot 12 mL into a separate 50 mL Falcon tube for each dish. Keep all aliquots at room temperature until use.

6. Remove the culture media from cells by gently pouring into the general waste beaker, then aspirate any remaining liquid.
7. Add 12 mL of freshly prepared 1.66 mM DSG solution to each 15 cm dish.

**CRITICAL:** If processing multiple dishes, stagger the addition of DSG (and all subsequent solutions) by 10–20 s per dish. Label each plate according to the order of processing to ensure consistent timing across all samples.

***Note:*** Pour gently to avoid detaching of cells, and cover each dish with a lid to prevent solution evaporation.

8. Incubate for 18 min at room temperature. Gently swirl dishes a few times during the incubation.

***Note:*** Crosslinking time may require re-optimization for different cell types.

9. During the DSG incubation, freshly prepare 1% formaldehyde solution by first diluting 16% or 37% methanol-free formaldehyde to 11% in FA dilution buffer, then diluting 1:11 in PBS.

**CRITICAL:** Formaldehyde is volatile and acutely toxic. Prepare solutions in a fume hood and wear appropriate personal protective equipment.

***Note:*** Prepare 12 mL of 1% formaldehyde solution per 15 cm tissue culture dish. To streamline handling, aliquot 12 mL into a separate 50 mL Falcon tube for each dish. Keep all aliquots at room temperature, with lids closed, until use.

10. Remove DSG solution into a general waste beaker and wash the cells three times with PBS by gentle pouring. Aspirate any remaining PBS after the final wash.
11. Add 12 mL of freshly prepared 1% formaldehyde to each 15 cm dish.

***Note:*** Pour gently to avoid detaching of cells, and cover each dish with a lid to prevent excessive solution evaporation.

12. Incubate for 8 min at room temperature. Gently swirl dishes a few times during the incubation.
13. Quench formaldehyde by adding 1.2 mL of 1.25 M glycine solution.

**CRITICAL:** Our recommended glycine amounts are sub-stoichiometric and allow some crosslinking to continue. For consistent results, ensure that both formaldehyde and glycine incubation times are completely consistent between all samples.

14. Incubate for 5 min at room temperature. Gently swirl dishes a few times during the incubation.
15. Remove formaldehyde solution into a dedicated FA waste container with a lid and wash the cells three times with ice-cold PBS by gentle pouring. Aspirate any remaining PBS after the final wash.

**CRITICAL:** Collect formaldehyde waste separately for proper disposal and keep the lid on the container at all times.

16. Add 3 mL of ice-cold PBS to each dish.
17. Scrape the cells and transfer to a 15 mL Falcon tube. Keep on ice.

***Note:*** If planning to process more than ∼24–36 samples in parallel (e.g. 2–3 chromatin factors across 4 conditions with 3 replicates), we recommend splitting each sample into multiple tubes before snap-freezing. This enables selective thawing and parallel processing of a manageable subset of chromatin factors at a time. Always process all conditions (and ideally replicates) of an experiment together; split factors across different experiment batches if necessary.

18. Centrifuge at 2000 × *g* for 7 min at 4°C; discard supernatant.
19. Flash-freeze cell pellets in liquid nitrogen and store at −80°C.

***Note:*** Snap freezing helps preserve sample integrity by preventing formation of ice crystals.

**Pause point:** Flash-frozen fixed cells may be stored at −80°C for several months.

### Shear chromatin and immunoprecipitate factors of interest

**Timing: 1 day**

This section describes how to extract and fragment chromatin from cells, then enrich factor-bound chromatin by immunoprecipitation.

20. Prepare the Covaris E220.
  a. Fill the tank with Milli-Q water until it reaches the ‘0’ mark indicated on the ‘fill’ side of the water tank.
  b. Degas the water. This takes 1 h.
  c. Cool water tank to 4°C. This takes 1 h.
  d. Set the centrifuge to 4°C.
21. Prepare LB1, LB2, LB3 buffers and BSA-PBS solution; store at 4°C.

**CRITICAL:** Sodium deoxycholate is light sensitive. Add to LB3 just before use, and minimize exposure to light.

**CRITICAL:** Perform all of the following steps on ice or at 4°C.

22. Briefly thaw cell pellets on ice.

**CRITICAL:** Thaw and process pellets from all conditions and biological replicates in parallel, provided the total number of samples is manageable. If processing in batches (see step 17), thaw the pellets from one batch and complete all steps through step 54 before proceeding to the next.

23. For each cell pellet (∼120 million cells from 6 × 15 cm dishes of HEK cells), add 40 mL LB1 buffer and resuspend the cells by rotating for 20 min at 4°C.

**CRITICAL:** If using a different cell number, scale LB1, LB2 and LB3 volumes accordingly.

24. Centrifuge at 1000 × *g* for 5 min at 4°C. Discard supernatant.
25. Resuspend pellets in 40 mL LB2 buffer and incubate for 5 min on ice.
26. Centrifuge at 1000 × *g* for 5 min at 4°C. Discard supernatant.
27. Resuspend pellets in 4 mL LB3 buffer on ice.

***Note:*** If working with smaller cell pellets, scale LB3 volumes according to cell number, then round to the nearest whole number (minimum of 1 mL). For example, if working with 40 million cells for IP of a single protein factor, resuspend the pellet in 1 mL of LB3 (rounded down from 1.33 mL).

28. Transfer 1 mL of chromatin to four separate Covaris milliTUBE 1 mL AFA Fiber tubes.

**CRITICAL:** Ensure that the final volume of each aliquot is exactly 1 mL. If there is a small amount of chromatin left over after aliquoting, it is better to discard it than overfill the AFA Fiber tubes.

***Note:*** Pipette gently to avoid generating bubbles.

29. Place tubes into the Covaris E220.

**CRITICAL:** When the Acoustic Assembly is at “Start Position”, carefully adjust the water level to the ‘0’ mark indicated on the ‘run’ side of the water tank. The water level should be 1 mm below the neck of each tube. Lower or higher water levels will reduce shearing efficiency.

**Figure 3:**
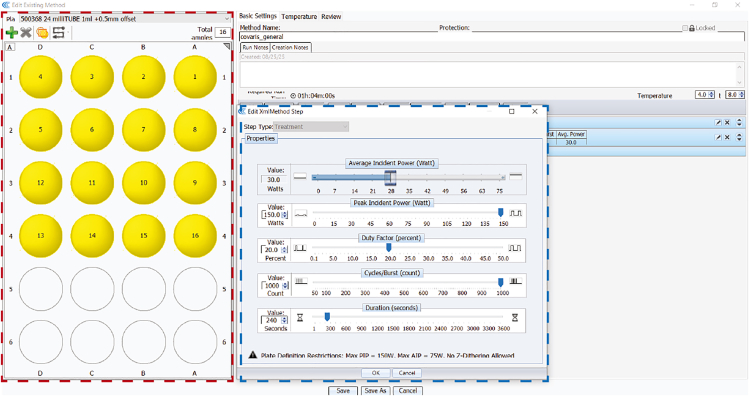
Example Covaris E220 ultrasonicator settings and tube layout Screenshot of the SonoLab software interface used to program the Covaris E220 focused-ultrasonicator. The sample tube layout in a 24-tube rack is shown on the left (outlined in red). On the right, the method editing window (outlined in blue) displays example acoustic shearing parameters, including duty factor, peak incident power, cycles per burst, and treatment duration.

30. Use the following settings to shear chromatin (also shown in Figure 3): peak incident power = 150, duty factor = 20%, cycles per burst = 1000, duration = 240 sec (per tube), temperature = 4°C.

***Note:*** These settings are optimized for HEK-293 double-crosslinked cells. If using an alternative shearing device, shearing tube size, or cell line, follow the steps described in ‘troubleshooting – problem 1’ to re-optimize fragmentation. Also see the ‘chromatin shearing’ subsection under materials and equipment setup.

***Note:*** To make the best use of time, we recommend washing beads and setting up antibody-bead conjugation (steps 39–42) during chromatin shearing. Use 2 mL Protein LoBind tubes.

31. Transfer sheared chromatin into microcentrifuge tubes and add 10% Triton X-100 to a final concentration of 1% (v/v). Invert gently to mix.
32. Centrifuge at 20,000 × *g* for 20 min at 4°C, then transfer the chromatin-containing supernatant into a new tube.
33. Measure the DNA concentration of each sample using a Qubit dsDNA High Sensitivity Assay Kit.

***Optional:*** Rapid de-crosslinking may improve the accuracy of concentration measurements, but in our experience, this is not necessary. If a more accurate measurement of DNA concentration is needed, remove FA crosslinks by incubating a small aliquot (e.g. 10 μL) of each chromatin sample with an equal volume of 2× decrosslinking buffer at 95°C for 10 min. Dilute in water and remeasure the concentration by Qubit. We do not recommend rapid decrosslinking for assessment of fragmentation efficiency, as heating to 95°C can fragment the DNA further, giving a false estimate of fragment size.^17^^,^^18^

***Optional:*** If using a spike-in for downstream data normalization, add spike-in chromatin to each sample after quantifying chromatin amounts. Maintain a consistent spike-in-to-sample chromatin ratio across all samples.

***Note:*** The optimal amount of spike-in chromatin depends on the antibody and target protein. For commonly used RNA Pol II antibodies, we typically use 1 μg of spike-in chromatin per 100 μg of sample chromatin (both measured by DNA content), aiming for approximately 5% of sequencing reads to originate from the spike-in.

34. Store a 20 μL aliquot (∼1.5%) of each chromatin sample at −20°C (input control).
35. For each IP, aliquot chromatin corresponding to 500 μg DNA into a new tube and adjust with LB3 + 1% Triton X-100 to a final volume of ∼1.5 mL (∼333 ng/μL). Keep both DNA amount and final volume identical across IPs.

***Note:*** The optimal amount of chromatin needed for each immunoprecipitation depends on the antibody efficiency and the abundance of the target protein crosslinked to chromatin. We recommend starting with chromatin obtained from 40 million cells for each IP (∼500 μg chromatin by DNA content). Downscale to 8 million cells (∼100 μg chromatin by DNA content) per IP if cost or cell material are limiting (see guidance in step 1).

36. In preparation for pre-clearing, wash 25 μL Protein A/G Dynabeads in BSA-PBS solution for each IP (3 × 200 μL washes).

**CRITICAL:** Ensure that beads are never left to dry. Remove the final wash from beads immediately before adding chromatin.

***Note:*** Use Protein LoBind tubes for steps 36–48.

37. To pre-clear samples, add each chromatin sample to a washed aliquot of protein A/G Dynabeads and rotate on a lab rotator at 12 rpm for 1 h at 4°C.
38. Place samples on a magnetic rack; transfer the supernatant to a new tube and discard the beads.
39. To prepare antibody-conjugated beads, wash 70 μL Protein A/G Dynabeads (which can bind 16.8 μg IgG) per sample in BSA-PBS solution (3 × 500 μL washes, 5 min each, rotating at 4°C).

***Note:*** Select Protein A, Protein G, or a 50:50 A/G mix according to their affinity for the antibody host species and IgG subtype, as recommended by the bead supplier. If the chosen antibody is not efficiently bound by either bead type, see troubleshooting – problem 6.

***Note:*** The amount of Protein A/G Dynabeads can be further optimized depending on factors such as target protein abundance, sample cell number, and antibody affinity.

40. Add 500 μL BSA-PBS solution to each washed bead aliquot and add 5–10 μg of the target antibody. If using a spike-in for downstream data normalization, add 1–2 μg of the spike-in antibody.

***Note:*** The precise antibody amount should be chosen based on the supplier datasheet or published ChIP-seq protocols using that antibody. If this information is not available, use 10 μg of antibody.

41. Incubate on a lab rotator at 12 rpm at 4°C for 1–2 h.
42. Wash antibody-bound beads in BSA-PBS solution (3 × 500 μL washes). For the last wash, incubate for 10 min on a lab rotator at 12 rpm at 4°C.
43. Resuspend antibody-bound beads in pre-cleared chromatin and incubate overnight at 4°C, rotating at 12 rpm on a lab rotator.

**CRITICAL:** Ensure that beads are never left to dry. Remove the final wash from antibody-conjugated beads immediately before adding chromatin. Alternatively, if processing speed is a concern, resuspend washed antibody-conjugated beads in a small volume of LB3, and add chromatin directly to the suspension.

### Purify DNA from immunoprecipitates

**Timing: 1 day and 2 h**

This section describes how to purify immunoprecipitated DNA from beads.

44. Prepare RIPA buffer and ChIP elution buffer; store at 4°C.

***Note:*** We use six washes with ice-cold RIPA buffer in place of the traditional stepwise low-salt, high-salt, and LiCl washes. RIPA combines high ionic strength with both ionic and non-ionic detergents to broadly remove nonspecific protein-protein and protein-DNA interactions in a single buffer. Sequential DSG + FA crosslinking stabilizes target complexes and helps them remain bound under these conditions, while non-crosslinked background is efficiently removed. This simplified approach reduces variability between replicates and yields consistently lower background without loss of target signal.

45. Place immunoprecipitation samples on a magnetic rack on ice and discard the supernatant.
46. Resuspend beads in 1 mL ice cold RIPA and transfer to a new Protein LoBind tube.
47. Wash Dynabeads six times with ice cold RIPA buffer, incubating samples on a turning wheel at 4°C for 5 min.
48. To elute DNA from the beads, place on a magnetic rack and carefully remove the supernatant. Then add 180 μL ChIP elution buffer and incubate for 1 h at 65°C with shaking at 1400 rpm.
49. After incubation, spin down at 200 × *g* for 1 min to collect liquid, and place the samples back on a magnetic rack. Transfer the DNA-containing supernatant into new tubes.
50. Thaw the input control sample (reserved at step 34) and adjust the volume to 180 μL with ChIP elution buffer.

***Optional:*** If RNA contamination is a concern, add 2 μL of RNase A (10 mg/mL) to each sample and incubate at 37°C for 30 min.

51. To digest proteins, add 20 μL 20 mg/mL Proteinase K to each sample (both eluted samples and input samples) and incubate overnight at 60°C with shaking at 1400 rpm.

**Pause point:** After protein digestion, decrosslinked samples may be stored at −20°C for several days.

52. Purify DNA from decrosslinked samples using the ChIP Clean & DNA Concentrator kit according to manufacturer’s instructions. Elute DNA with 11 μL elution buffer (10 mM Tris-Cl, pH 8.5).

**CRITICAL:** Ensure the elution buffer is free of EDTA; avoid using buffers such as TE (10 mM Tris-Cl, 1 mM EDTA, pH 8.0).

***Note:*** Elution volumes can be adjusted for compatibility with the chosen library preparation kit.

53. Use 1 μL of each sample to quantify DNA using a Qubit dsDNA High Sensitivity Assay Kit. A total yield of 10–20 ng DNA or more indicates a successful IP and is sufficient to generate high-quality sequencing libraries.

***Note:*** If necessary, library preparation can be performed with as little as 500 pg of DNA, though this may impact the quality of the data.

54. Assess shearing efficiency with input control.
  a. Dilute 1 μL of input control DNA 1:5 in nuclease-free water.
  b. Run 1 μL on an Agilent TapeStation System using a D5000 ScreenTape, or on an Agilent Bioanalyzer with a High Sensitivity DNA kit.

***Note:*** To avoid unnecessary loss of immunoprecipitated material, we recommend assessing fragment size using input control samples only. Successful chromatin shearing typically yields a single peak around 200 bp, with at least 85% of fragments ranging between 100 and 2000 bp.

***Note:*** In principle, diagnostic ChIP-qPCR at established target and control loci can be useful at this step, as it provides a rapid readout of IP efficiency before proceeding to library preparation. However, in our experience there are two limitations to this approach. First, for challenging or novel chromatin-associated proteins, well-characterized positive control loci and validated qPCR primer sets are often not available, which restricts the use of a diagnostic qPCR check. Second, ChIP-qPCR requires consumption of a non-trivial amount of the recovered ChIP DNA, which can be limiting when working with low-abundance factors. For these reasons, we recommend proceeding directly to library preparation without an intermediate ChIP-qPCR step.

**Pause point:** DNA samples may be stored at 4°C for less than a week, or at −20°C for several months.

### Prepare libraries and set up next-generation sequencing run

**Timing: 2 days**

This section describes the recommended conditions for building Illumina-compatible libraries and setting up the sequencing run.

55. Use 10–50 ng of purified DNA to prepare libraries for high-throughput sequencing, following the manufacturer’s guidelines. For input control samples, use 50 ng of DNA.

***Note:*** Any standard library preparation protocol with Illumina-compatible index primers can be used. For instance, you can use the NEBNext Ultra II DNA Library Prep Kit for Illumina (NEB) together with NEBNext Multiplex Oligos. No further DNA fragmentation is required during library preparation.

***Note:*** In our experience, libraries made from more than 5 ng of pulled-down DNA provide the best results. However, as little as 500 pg of DNA should be enough to successfully prepare libraries for sequencing.

**CRITICAL:** Adjust the number of PCR cycles to the amount of starting DNA material, following the manufacturer’s guidelines. Use a minimum of 5 PCR cycles for all samples, including input control DNA, to ensure an appropriate yield. Otherwise, keep the PCR cycle number as low as possible, and do not exceed 14 cycles.

56. Use a Qubit dsDNA High Sensitivity Assay Kit to quantify each DNA library. Assess fragment size distribution by running 1 μL of the library on an Agilent Bioanalyzer (High Sensitivity DNA kit) or an Agilent TapeStation System (D1000 or D5000 ScreenTape).

***Note:*** With the NEBNext Ultra II DNA Library Prep Kit, successful dxChIP-seq libraries should show a broad peak ranging from 250–600 bp and a mean fragment size of ∼500 bp; adaptor dimers (< 200 bp) should be minimal or absent.

**Pause point:** DNA samples may be stored at 4°C for less than a week, or at −20°C for several months.

57. Pool indexed libraries for sequencing. Typically, samples are pooled at a total concentration of 10 nM, diluted in 10 mM Tris-HCl (pH 8.5) as necessary.

***Note:*** Calculate molarity using the Qubit-measured DNA concentration and the average fragment length from the Bioanalyzer or TapeStation:

$$M\;\left(\text{nM}\right)=\left[\text{DNA}\;\text{concentration}\;\left(\text{ng}/\mu L\right)\times 10^{6}\right]/\left[660\times \text{average}\;\text{fragment}\;\text{length}\;\left(\text{bp}\right)\right]$$

**CRITICAL:** Mix libraries at equal molarity. Uneven pooling will skew the read depth across samples. See Table S1 for detailed guidance on pooling calculations.

**Pause point:** The 10 nM master pool can be stored at −20°C for several weeks without loss of quality.

58. Dilute pooled libraries in the resuspension buffer supplied by the sequencing platform to be used (for example, NextSeq 1000/2000 Resuspension Buffer for the NextSeq 2000) to the recommended final concentration (488 pM is the standard recommendation for NextSeq 2000 XLEAP-SBS chemistry kits, though other kits have different requirements). Sequence libraries in paired-end mode using a kit providing 100 sequencing cycles or more.

**CRITICAL:** The sequencing depth required will depend on the target protein; RNA Pol II or histone marks with a broad genomic distribution require a larger number of reads for an appropriate coverage (∼40–60 million reads per replicate), while localized targets such as transcription factors require fewer reads (∼20 million reads per replicate).

### Align and process reads using standard software

**Timing: variable**

Data analysis should follow a typical ChIP-seq pipeline, incorporating spike-in normalization if applicable.

59. Set up a working directory and download a reference genome of the target species.

# Navigate to desired working directory

cd Working_directory

# Create output folders

mkdir Genomes qc SAM BAM BAM_dup BW Tiles Bed Coverage Plots

# Download reference genome and gene annotation

wget ftp://ftp.ensembl.org/pub/release-102/fasta/homo_sapiens/dna/Homo_sapiens.GRCh38.dna.primary_assembly.fa.gz -O ./Genomes/Homo_sapiens.GRCh38.dna.primary_assembly.fa.gz

wget ftp://ftp.ensembl.org/pub/release-102/gtf/homo_sapiens/Homo_sapiens.GRCh38.102.gtf.gz -O ./Genomes/Homo_sapiens.GRCh38.102.gtf.gz

# Decompress reference genome and gene annotation

gunzip ./Genomes/Homo_sapiens.GRCh38.dna.primary_assembly.fa.gz

gunzip ./Genomes/Homo_sapiens.GRCh38.102.gtf.gz

60. Trim and quality filter reads with Trim Galore, using a quality threshold of 30.

# Decompress fastq files (note: these may reside in an external directory)

gunzip /fastq_dir/sample_R1.fastq.gz

gunzip /fastq_dir/sample_R2.fastq.gz

# Trim adapters and quality filter reads

trim_galore -q 30 --fastqc --paired --output_dir ./qc /fastq_dir/sample_R1.fastq /fastq_dir/sample_R2.fastq

61. Align reads to the target genome using Bowtie2 (default parameters).

# Build index

bowtie2-build -f ./Genomes/Homo_sapiens.GRCh38.dna.primary_assembly.fa ./Genomes/Homo_sapiens.GRCh38.dna.primary_assembly.bt2

# Align reads to target genome

bowtie2 --end-to-end -x ./Genomes/Homo_sapiens.GRCh38.dna.primary_assembly.bt2 -1 ./qc/sample_R1_val_1.fq -2 ./qc/sample_R2_val_2.fq -S ./SAM/sample.sam

# Sort and index SAM files

samtools sort ./SAM/sample.sam -o ./BAM/sample.bam

samtools index ./BAM/sample.bam

***Note:*** If samples contain a spike-in, concatenate the target (e.g. human) and spike-in (e.g. *D. melanogaster*) reference genomes, build a combined index, then align reads to the combined index file. Before proceeding to the next step, count the number of reads that uniquely map to the spike-in reference genome. After counting, discard all reads that align to the spike-in reference genome and proceed to step 62.

62. Mark and remove PCR duplicates with Picard (default parameters).

# Mark and remove PCR duplicates

java -jar picard.jar MarkDuplicates I=./BAM/sample.bam O=./BAM_dup/sample.bam REMOVE_DUPLICATES=true M=./BAM_dup/sample.metrics.txt

# Index BAM file

samtools index ./BAM_dup/sample.bam

63. Generate RPKM-normalized bigwig files using deepTools.

bamCoverage --bam ./BAM_dup/sample.bam -o ./BW/sample.25nt.RPKM.bw -of bigwig --binSize 25 --normalizeUsing RPKM

***Note:*** If samples contain a spike-in, calculate normalization factors by dividing any constant (e.g. 10,000,000) by the total number of reads that align to the spike-in genome. Replace “--normalizeUsing RPKM” with “--scaleFactor X” (where ‘X’ is the calculated normalization factor) in the bamCoverage command.

***Optional:*** We find that a resolution of 25 bp (specified by ‘--binSize 25’) typically provides the best balance for most dxChIP-seq data. However, for new datasets or protein targets, consider testing multiple resolutions then selecting the most appropriate by visual inspection on IGV.

**Figure 4:**
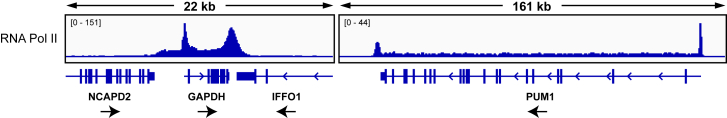
Representative dxChIP-seq signal at control genes RPKM-normalized total RNA Pol II (D8L4Y) signal in wild-type HEK-293 cells, generated using the alignment and processing codes in this protocol.

64. Manually inspect the data in IGV to verify quality (Figure 4).

### Perform metagene analysis

**Timing: 30 min**

Metagene analysis is helpful for evaluating dataset quality and can provide insights into the mechanisms under investigation. For example, the localization of profiled chromatin factors relative to genic features such as transcription start sites (TSSs), gene bodies and transcription termination sites (TTSs) is often central to understanding how they function. Equally, the same approach can be adapted to other features such as enhancers, DNA replication origins and common fragile sites.

65. Pre-process dxChIP-seq data for mapping.

# Convert bigwig file into bed file

bigWigToBedGraph ./BW/sample.25nt.RPKM.bw ./Bed/sample.25nt.RPKM.bed

# Sort bed file for compatibility with bedtools suite

sort -k1,1 -k2,2n ./Bed/sample.25nt.RPKM.bed > ./Bed/sample.25nt.RPKM.sorted.bed

66. Construct a tiled gene annotation file in R.

# Set working directory

setwd("∼/Working_directory")

# Load necessary libraries

library(rtracklayer)

library(dplyr)

library(ggplot2)

# Disable scientific notation to prevent errors in the code

options(scipen = 999)

# Read the GTF file into R

hg38.annotations <- import("./Genomes/Homo_sapiens.GRCh38.102.gtf")

# Convert it into a data frame and filter for genes; to prevent errors, set the minimum gene width to the number of tiles that each gene will be split into. Depending on the goals of the analysis, it may be appropriate to add further filters for gene biotype, width or minimum coverage in this step.

hg38.genes <- hg38.annotations %>% as.data.frame() %>% filter(type == "gene") %>% select(seqnames, start, end, width, strand, gene_biotype, gene_name) %>% filter(width>60)

# Filter genes by strand

hg38.genes.pos <- hg38.genes %>% filter(strand=="+")

hg38.genes.neg <- hg38.genes %>% filter(strand=="-")

# Define upstream and downstream flanking regions

# Note that mutate() updates columns from left to right: it first moves ‘start’ 5 kb upstream, then sets ‘end’ 5 kb downstream of this new start. The resulting window (for upstream flanking regions) therefore runs from −5 kb to the TSS.

hg38.genes.pos.upstream.flank <- hg38.genes.pos %>% mutate(start=start-5000,end=start+5000) %>% filter(start>0)

hg38.genes.neg.upstream.flank <- hg38.genes.neg %>% mutate(start=end,end=start+5000)

hg38.genes.pos.downstream.flank <- hg38.genes.pos %>% mutate(start=end,end=start+5000)

hg38.genes.neg.downstream.flank <- hg38.genes.neg %>% mutate(start=start-5000,end=start+5000) %>% filter(start>0)

# Save files

write.table(hg38.genes.pos, "./Tiles/hg38.genes.pos.bed", sep = "\t", quote = F, col.names = F, row.names = F)

write.table(hg38.genes.neg, "./Tiles/hg38.genes.neg.bed", sep = "\t", quote = F, col.names = F, row.names = F)

write.table(hg38.genes.pos.upstream.flank, "./Tiles/hg38.genes.pos.upstream.flank.bed", sep = "\t", quote = F, col.names = F, row.names = F)

write.table(hg38.genes.neg.upstream.flank, "./Tiles/hg38.genes.neg.upstream.flank.bed", sep = "\t", quote = F, col.names = F, row.names = F)

write.table(hg38.genes.pos.downstream.flank, "./Tiles/hg38.genes.pos.downstream.flank.bed", sep = "\t", quote = F, col.names = F, row.names = F)

write.table(hg38.genes.neg.downstream.flank, "./Tiles/hg38.genes.neg.downstream.flank.bed", sep = "\t", quote = F, col.names = F, row.names = F)

# Split genes into 60 tiles and flanking regions into 20 tiles

hg38.genes.pos.60t <- fread("bedtools makewindows -b ./Tiles/hg38.genes.pos.bed -n 60 -i winnum")

hg38.genes.neg.60t <- fread("bedtools makewindows -b ./Tiles/hg38.genes.neg.bed -n 60 -i winnum -reverse")

hg38.pos.upstream.flank.20t <- fread("bedtools makewindows -b ./Tiles/hg38.genes.pos.upstream.flank.bed -n 20 -i winnum")

hg38.neg.upstream.flank.20t <- fread("bedtools makewindows -b ./Tiles/hg38.genes.neg.upstream.flank.bed -n 20 -i winnum -reverse")

hg38.pos.downstream.flank.20t <- fread("bedtools makewindows -b ./Tiles/hg38.genes.pos.downstream.flank.bed -n 20 -i winnum")

hg38.neg.downstream.flank.20t <- fread("bedtools makewindows -b ./Tiles/hg38.genes.neg.downstream.flank.bed -n 20 -i winnum -reverse")

# Combine positive and negative strand tiles, and adjust bin numbers for sequential coverage across each gene

hg38.genes.60t <- rbind(hg38.genes.pos.60t, hg38.genes.neg.60t) %>% mutate(V4 = V4 + 20)

hg38.upstream.flank.20t <- rbind(hg38.pos.upstream.flank.20t, hg38.neg.upstream.flank.20t)

hg38.downstream.flank.20t <- rbind(hg38.pos.downstream.flank.20t, hg38.neg.downstream.flank.20t) %>% mutate(V4 = V4 + 80)

# Combine tiles from genes and flanking regions

hg38.genes.and.flanks <- rbind(hg38.genes.60t, hg38.upstream.flank.20t, hg38.downstream.flank.20t)

# Save annotation file

write.table(hg38.genes.and.flanks, "./Tiles/hg38.genes.and.flanks.bed", sep = "\t", quote = F, col.names = F, row.names = F)

# Sort annotation file for compatibility with bedtools suite

system("sort -k1,1 -k2,2n ./Tiles/hg38.genes.and.flanks.bed > ./Tiles/hg38.genes.and.flanks.sorted.bed")

***Note:*** When analyzing a single genomic site - such as a TSS, replication origin, or site of DNA damage - it is unnecessary to define separate windows for upstream and downstream flanks. Instead, create a symmetric window of 1–20 kb around the midpoint of the feature and divide it into 100 tiles. Plot as described in step 67, adjusting x-axis breaks and labels as needed.

**CRITICAL:** Ensure that both the window size and normalization method are appropriate for the feature being studied. For example, metagene plots centered on TTSs may require a larger window (∼10 kb upstream and downstream) and normalization to the last bin (rather than the first) in step 67. These adjustments allow for accurate comparison of chromatin factors at TTSs while avoiding inappropriate normalization to the gene body, regions of readthrough transcription, or inaccurately annotated termination sites.

**Figure 5:**
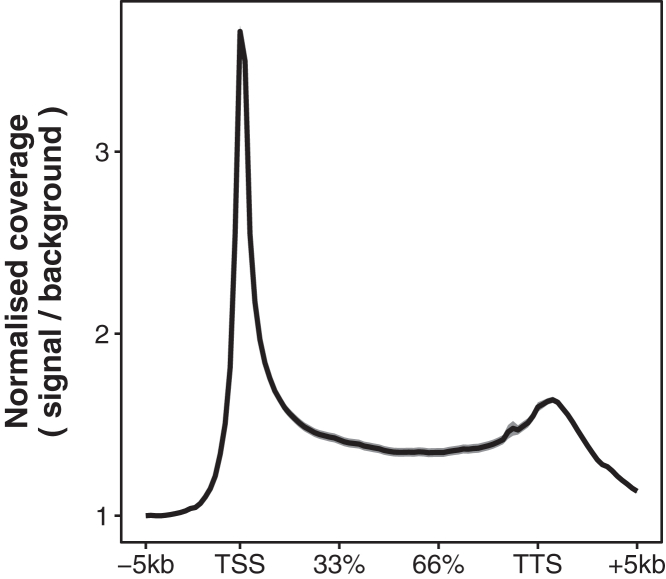
Example dxChIP-seq metagene Metagene of total RNA Pol II (D8L4Y) occupancy in wild-type HEK-293 cells, generated using the plotting codes in this protocol. Data are represented as mean ± SEM.

67. Map dxChIP-seq coverage at tiled genes and construct a metagene plot in R, normalizing to upstream intergenic regions (Figure 5).

# Map dxChIP-seq signal at tiled genes

sample.coverage <- fread("bedtools map -a ./Tiles/hg38.genes.and.flanks.sorted.bed -b ./Bed/sample.25nt.RPKM.sorted.bed -c 4 -o mean -null 0")

# Rename columns

colnames(sample.coverage) <- c("chr", "start", "end", "bin", "COV")

# Take mean signal at each coordinate; normalize to the mean signal at the upstream intergenic flank

sample.plot <- sample.coverage %>% group_by(bin) %>% summarise(COV.mean = mean(COV),SEM = sd(COV)/sqrt(n())) %>% mutate(COV.norm = COV.mean/first(COV.mean), SEM.norm = SEM/first(COV.mean))

# Create plot

plot <- sample.plot %>% ggplot(aes(x = bin, y = COV.norm)) +

 geom_line(size = 1) +

 geom_ribbon(aes(ymin = COV.norm - SEM.norm, ymax = COV.norm + SEM.norm), alpha = 0.5, color = NA) +

 scale_x_continuous(breaks = c(1,20,40,60,80,100), labels = c("1" = "-5kb", "20" = "TSS", "40" = "33%", "60" = "66%", "80" = "TTS", "100" = "+5kb")) +

 ylab("Normalised coverage\n( signal / background )") +

 theme_bw() +

 theme(

 aspect.ratio = 1,

 axis.text.x = element_text(size = 11, hjust = 0.5),

 axis.text.y = element_text(size = 11),

 axis.title.x = element_blank(),

 axis.title.y = element_text(size = 12, face = "bold", vjust = 4),

 plot.title = element_text(size = 15, face = "bold"),

 panel.grid.major = element_blank(),

 panel.grid.minor = element_blank())

# Save plot

pdf("./Plots/sample.plot.pdf", width=4, height=3.5)

plot

dev.off()

***Note:*** If bigwigs have been normalized using a spike-in, additional normalization to the upstream intergenic signal is not required. In the plotting function, use mean coverage instead of normalized coverage (‘COV.mean’ instead of ‘COV.norm’; ‘SEM’ instead of ‘SEM.norm’).

## Expected outcomes

A successful dxChIP-seq experiment produces high-complexity libraries that yield clear genome-wide maps of chromatin factor binding. In the example of total RNA Pol II, when visualized in a genome browser, these display sharp peaks at TSSs, consistent occupancy across gene bodies, and a smaller peak at TTSs. Metagene plots typically confirm these patterns and are particularly useful for revealing condition-dependent shifts in RNA Pol II distribution across gene units.

The enhanced sensitivity of dxChIP-seq allowed us to uncover subtle changes in RNA Pol II occupancy and phosphorylation that are difficult to detect with conventional ChIP-seq. For example, following treatment with transcriptional inhibitor triptolide, dxChIP-seq reveals not only the expected global reduction in RNA Pol II levels, but also a 55 bp upstream shift corresponding to relocalization of RNA Pol II signal from the pausing site to the TSS.^1^ Similarly, perturbations of transcriptional regulators such as CUL3-ARMC5 or INTS8 uncover distinct changes in RNA Pol II occupancy at promoter-proximal regions and across gene bodies, allowing detailed analysis of their roles in transcriptional regulation. We have also successfully used dxChIP-seq to profile the genomic occupancy of a number of transcription regulators, many of which are difficult targets that are low abundance or do not bind DNA directly (unpublished data).

Overall, dxChIP-seq provides a straightforward modification to standard ChIP-seq workflows, enabling sensitive detection of chromatin occupancy across a much broader range of factors. It is particularly useful for profiling proteins that do not bind DNA directly but are recruited to chromatin through interaction partners (e.g. SMAD3^4^), and also improves the signal-to-noise ratio when applied to DNA-bound proteins such as histones or RNA Pol II.^1^^,^^4^

## Limitations

Like other ChIP-seq methods, dxChIP-seq does not reveal strand specificity or whether a protein is functionally active. Depending on the context, it may be necessary to complement it with methods that measure activity, such as transient transcriptome sequencing (TT-seq) or precision run-on sequencing (PRO-seq) in transcription studies.

## Troubleshooting

### Problem 1

**Figure 6:**
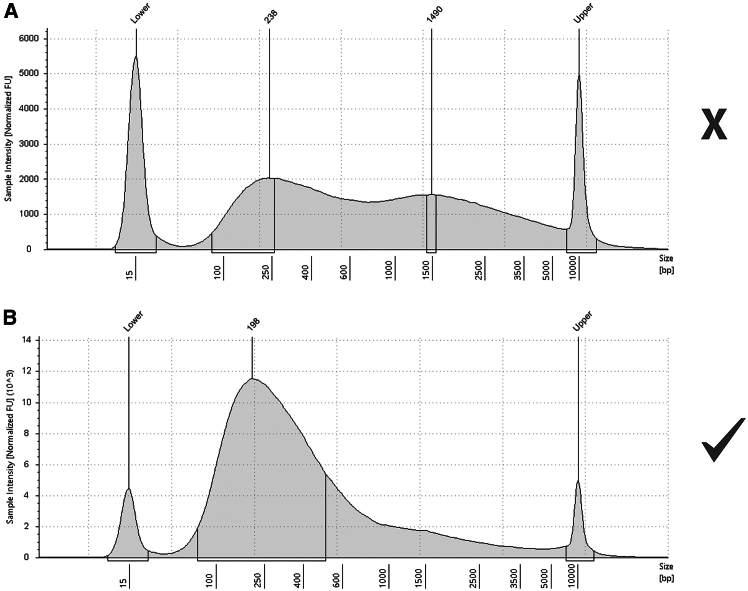
Optimization of shearing conditions DNA size profiles of inadequately (A) and adequately (B) sheared DNA, analyzed using an Agilent TapeStation D5000 assay.

Large fragment sizes after shearing (Figure 6A).

### Potential solution

Sub-optimal chromatin shearing can lead to fragments of inappropriate size for sequencing, low resolution of data, or a failed IP due to decreased protein stability. If fragment sizes are too large with our recommended parameters, or when using a new cell line, follow these steps to re-optimize shearing conditions.

• **Prepare crosslinked samples:** Generate one single-crosslinked (8 min 1% formaldehyde) and one double-crosslinked (18 min 1.66 mM DSG + 8 min 1% formaldehyde) cell pellet using the “double crosslinking” protocol. Proceed with the protocol until samples are ready for shearing (step 29).
• **Optimize shearing efficiency and protein stability:** Shear single- and double-crosslinked samples using standard conditions (PIP=150, CPB=1000, Duty=20, Temp=4°C) for a total of 10 min, pausing every 2 min to take a 10 μL aliquot (resulting in a series of samples: 2 min, 4 min, 6 min, 8 min, 10 min shearing). Replace the lost volume with 10 μL LB3 buffer.
  ○ Use double-crosslinked samples to test DNA size distribution. Decrosslink and purify DNA (steps 50–52, as described for “ChIP input” samples), then assess fragment size by agarose gel electrophoresis or a similar method. If using an Agilent TapeStation System, run 1 μL on a D5000 ScreenTape. Acceptable shearing times will yield a high proportion of 200–300 bp fragments; at least 85% should be between 100 and 2000 bp (Figure 6B).
  ○ Use single-crosslinked samples to test protein stability (double-crosslinked samples contain stable protein-protein crosslinks that alter electrophoretic mobility of individual proteins and cannot be used for western blot). Add 10 μL 2× Laemmli buffer, boil for 5 min, then perform a western blot using an antibody detecting the factor of interest. With increased shearing time, proteins may denature and precipitate out of the solution. Acceptable shearing times will not destroy more than 50% or protein signal on the western blot.
  ○ An ideal shearing time for the subsequent experiments is chosen as a compromise between a. and b. – yielding appropriately sized DNA fragments without causing excessive loss of the target protein.
• **Adjust crosslinking conditions:** If large fragment sizes persist across all time points, or suitable fragment sizes appear only at highly sheared time points where the protein factor of interest is unstable, it is likely that the sample is over-crosslinked. Crosslink cells with shorter incubation times (e.g. 10 min 1.66 mM DSG + 8 min 1% formaldehyde), then reassess shearing efficiency and factor stability.

### Problem 2

Low DNA recovery (< 1 ng) after IP, or a flat signal with no peaks in IGV.

### Potential solution

Libraries can be prepared with as little as 500 pg DNA, although this may reduce data quality. If low-input library preparation fails, or the resulting data gives a ‘flat’ IGV signal after sequencing and processing, it is likely that the immunoprecipitation did not work properly. If using an unvalidated antibody, consider switching to a different one and repeating the experiment. Ensure thorough verification of the bead-antibody combination and inclusion of proteinase inhibitors (and phosphatase inhibitors if studying specific phosphorylation marks) in all lysis and immunoprecipitation buffers. If no technical issues are identified, we recommend testing different chromatin input concentrations (step 35) then repeating the experiment with the amount that yields the highest DNA recovery. If no suitable antibodies are available, we recommend adding a tag (e.g. V5) to the factor of interest through CRISPR knock-in, then performing dxChIP-seq using a tag-specific antibody.

### Problem 3

The alignment and data processing steps are too slow.

### Potential solution

To speed up data processing, make use of multi-threading based on the available resources of your operating system or high-performance computing (HPC) cluster. Most recommended processing software supports multi-threading, though Picard does not, and trim-galore is limited to a maximum of 9 cores. The number of cores can be specified in each command, depending on available resources. For example, if your HPC cluster has 112 available cores, specify the following options during data processing.

• trim_galore: --cores 9.
• bowtie2-build: --threads 112.
• bowtie2: --threads 112.
• samtools sort: -@ 112 -m 2G.
• samtools index: -@ 112.
• bamCoverage: -p 112.

### Problem 4

The signal-to-background ratio is low / data quality is uncertain.

### Potential solution

If you are unsure about the data quality or suspect high background noise, perform additional quality control checks to confirm whether the experiment was successful. If any of these checks fail, consider repeating the experiment.

• **Assess the proportion of multi-mapping reads:** >70% uni-mapping is typical; <50% raises concerns.
• **Check the Pearson correlation between replicates:** >0.9 indicates high reproducibility; 0.3–0.4 indicates no correlation.
• **Perform visual inspection in IGV:** for example, RNA Pol II signal should be significantly enriched at genes and show sharp peaks at TSSs with minimal signal in intergenic regions.

### Problem 5

The metagene plots are noisy.

### Potential solution

If the dxChIP-seq dataset is of sufficient quality but metagene clarity needs improvement, we recommend modifying the following steps during data processing.

• **Filter out ‘noisy’ types of genes:** We find that non-coding RNAs and short protein coding genes can introduce noise into metagene plots. By limiting the analysis to protein-coding genes longer than 1000 bp, metagene quality may be improved. This can be achieved by modifying the following line of code in step 66 (suggested edits are bolded):

hg38.genes <- hg38.annotations %>% as.data.frame() %>% filter(type == "gene") %>% select(seqnames, start, end, width, strand, gene_biotype, gene_name) %>% filter(**gene_biotype=="protein_coding",** width>**1000**)

• **Identify and remove outliers:** A single outlier can sometimes introduce a ‘spike’ in the metagene. If the issue persists after filtering out non-coding RNAs and short genes, examine the coverage table (‘sample.coverage’ in step 67) for significant outliers. Cross-check the bin number of potential outliers with the location of the spike in the x-axis of the metagene, then manually inspect the region in IGV. If removal can be justified, filter the causative gene out of the analysis by modifying the following line of code in step 66 (suggested edits are bolded):

hg38.genes <- hg38.annotations %>% as.data.frame() %>% filter(type == "gene"**, gene_name != "PROBLEMATIC_GENE_NAME"**) %>% select(seqnames, start, end, width, strand, gene_biotype, gene_name) %>% filter(gene_biotype=="protein_coding", width>1000)

• **Exclude problematic genomic regions:** Repetitive genomic regions can accumulate multi-mapping reads that interfere with metagene analysis in some datasets. To remove these reads, we suggest adding two further data-processing steps:
  ○ Remove the ENCODE blacklist from the mapping file after step 65:
    # Download the hg38 ENCODE blacklist
    wget -O ./Genomes/hg38.blacklist.bed.gz https://github.com/Boyle-Lab/Blacklist/raw/master/lists/hg38-blacklist.v2.bed.gz
    # Unzip the blacklist file
    gunzip ./Genomes/hg38.blacklist.bed.gz
    # Format the blacklist file so that it is consistent with the mapping file
    awk '{sub(/ˆchr/, "", $1); print $1 "\t" $2 "\t" $3}' ./Genomes/hg38.blacklist.bed > ./Genomes/hg38.blacklist.2.bed
    # Subtract the ENCODE blacklist from the mapping file
    bedtools subtract -a ./Bed/sample.50nt.RPKM.sorted.bed -b ./Genomes/hg38.blacklist.2.bed > ./Bed/sample.50nt.RPKM.sorted.blacklist.bed
  ○ Remove multi-mapping reads after step 62:
    # Filter out low quality (including multi-mapping) reads
    samtools view -bq 1 ./BAM_dup/sample.bam > ./BAM_dup/sample.unique.bam
    # Index the filtered BAM file
    samtools index ./BAM_dup/sample.unique.bam

### Problem 6

Protein A and Protein G have low affinities for the species and immunoglobulin subtype of the chosen antibody.

### Potential solution

Certain species and immunoglobulin subtypes are poorly bound by both Protein A and Protein G. For example, commonly used antibodies against phosphorylated residues on the RNA Pol II C-terminal domain are a rat IgG1 isotype, which is not efficiently captured by either bead type. To overcome this, we recommend pre-incubating washed Protein A or G beads with a saturating amount of a bridging antibody between steps 39 and 40. For rat IgG1, we typically pre-incubate washed Protein G beads with 40 μg of rabbit anti-rat IgG (Abcam, ab6703) for at least 1 h. Beads are then washed before proceeding with conjugation of the factor-specific antibody as usual.

## Resource availability

### Lead contact

Further information and requests for resources and reagents should be directed to and will be fulfilled by the lead contact, Ana Tufegdžić Vidaković (atv@mrc-lmb.cam.ac.uk).

### Technical contact

Technical questions on executing this protocol should be directed to and will be answered by the technical contacts, Ana Tufegdžić Vidaković (atv@mrc-lmb.cam.ac.uk) (experimental) and Andrew Zeller (azeller@mrc-lmb.cam.ac.uk) (bioinformatic analysis).

### Materials availability

This study did not generate any new materials.

### Data and code availability

This study did not generate any new datasets or code.

## Acknowledgments

A.Z. is supported by a PhD fellowship from the Boehringer Ingelheim Fonds and a Neuberger Studentship from the Max Perutz Fund in collaboration with Trinity College. A.T.V. and J.E.S. are supported by a core grant to the LMB from the Medical Research Council (refs. MC_UP_1201/28 and U105178808, respectively). I.S. is supported by a César Milstein Studentship from the Darwin Trust of Edinburgh. S.J.V. is supported by a Rubicon fellowship (NWO, 019.161LW.017), NHMRC EL1 fellowship (GNT1178339), a CSL Centenary Fellowship, and a Snow Medical Fellowship. We thank MRC LMB Facilities for supporting our work (Flow Cytometry, Mechanical and Electronics Workshops, and Scientific Computing), and Shraddha Nayak (LMB Visual Aids) for valuable feedback on data visualization.

## Author contributions

A.T.V. conceptualized and supervised the study; A.T.V. and S.J.V. performed initial optimization of the protocol; A.Z., I.S., R.C., and Y.B. performed further experiments contributing to the study; R.C. and Y.B. generated the final datasets; A.Z. analyzed the data; A.Z., A.T.V., A.C., I.S., R.C., and Y.B. wrote and edited the manuscript; A.T.V. and A.Z. were responsible for project administration; A.T.V. and J.E.S. were responsible for supervision and funding acquisition.

## Declaration of interests

The authors declare no competing interests.

## Supplemental information

Table S1. Sample Pooling Calculator
